# Introducing work, welfare, and qualitative studies of health

**DOI:** 10.3402/qhw.v9.23800

**Published:** 2014-03-14

**Authors:** Mikael Jonasson

**Affiliations:** Department of Social- and Health Sciences, Halmstad University

Halmstad University's profile as the University of Innovation consists of the strong area of Health and Lifestyle. As many European societies are characterized by societal challenges, this strong area will turn out to be an area of research that has strong future relevance. Challenges related to an aging population, increased mental illness among young people, marginalization of disabled people, and the issues related to working life and health will be monitored by researchers within work and welfare with the help of qualitative studies.

Although indicated as divided, the research areas of welfare and working life are intimately intertwined because welfare systems need to be organized. As welfare systems are based on the fact that health and well-being are dependent on organizational principles of stable structures that enable individual freedom and equal opportunities, the opposite of organizing will probably lead to health problems, both within a wider society and within organizations. Thus, the division work and welfare focuses on innovative approaches to health and well-being from a structural, organizational, and individual perspective. The division has an interdisciplinary approach with research in fields such as welfare and education systems and their impact on vulnerable groups, different aspects of working life and work organization, and culture and social life (Jonasson, 2013; Tideman, 2008). The division embraces knowledge from different academic disciplines such as disability studies, social work, social psychology, human geography, social medicine, and work science. The aim of the work and welfare division is to encourage the development of a better understanding of the impact of social conditions and processes concerning work and welfare on health and well-being.

All types of organizations create different conditions for health and well-being. The research concerning work, health, and well-being will continue to explore the relation between the organizations of humans, and the health and well-being consequences from an individual, group, and society level. In different words, *it involves human existence through social and embodied and subjective experiences that affect health and well-being* (see Merleau-Ponty, 2002, p. 45). The research will also include people that, because of disabilities, often stand far from the center of society. These groups experience different kinds of challenges that also produce consequences for health and wellbeing that need to be rediscovered again and again, in order to be brought up front from the sharp boundaries of normality. The grounding of research of humans, independent of whether they are organized within a welfare society or within a public health organization, will necessarily have to be involved in interpretation of experienced meaning, perspectives, and positions. It demands, what Hallberg (2013) calls, a perspective of others.

Theoretical and methodological development concerning work, well-being, and health will also be developed within this division of the journal. It involves observations, interviews, action research, and ongoing as well as summative evaluation approaches, many of which may be placed within the traditions of phenomenology, ethnomethodology, and wider qualitative methods (Garfinkel, 1967; Goffman, 1970; Silverman, 1993). The fundamental question within ethnomethodology is to observe through the wide question: “What is going on there?”. In that sense, the methodology has intimate analytical connections with “grounded theory,” where the resistances toward preconceived categories are similar, and where theories tend to produce complex, rather than simplified worlds (Glaser & Strauss, 1967; Turner, 1983). Thus, in order to strengthen the inherent limitations of explanatory powers in grounded theory, it is necessary that qualitative methods, emergent or “discovered” theory, and rationalistic forms of proof and theorizing may proceed hand in hand (Layder, 1982, pp. 33–34). The question of emergent theory, as an important issue in grounded theory, as well as in phenomenology and ethnomethodology, is thus also the recognition of the intimately related approach of allowing discovery of emergent worlds, independent of whether they are the patients, employers at a company, or marginalized persons in society.

Qualitative studies of work, welfare, and health also aspire to participate with research that propels innovations and can be utilized in response to future societal challenges. The division of work and welfare has committed to the university strategy for innovations. Research concerning work and welfare will therefore include focus on humans, work, welfare, marginalized groups, organizations, and health and life styles, where qualitative approaches will be important for producing research that is of benefit for society.

*Mikael Jonasson* Department of Social- and Health Sciences, Halmstad University

## References

[CIT0001] Glaser B, Strauss A (1967). The Discovery of Grounded Theory.

[CIT0002] Goffman E (1970). *Interaction ritual*. [När människor möts. Studiet av det direkta samspelet mellan människor].

[CIT0003] Hallberg L (2013). Editorial—Quality criteria and generalization of results from qualitative studies. International Journal of Qualitative Studies on Health and Well-Being.

[CIT0004] Jonasson M (2013). The AKKA-board–performing mobility, disability and innovation. Disability & Society.

[CIT0005] Layder D (1982). Grounded theory: A constructive critique. Journal for the Theory of Social Behaviour.

[CIT0006] Merleau-Ponty M (2002). Phenomenology of perception.

[CIT0007] Silverman D (1993). Interpreting qualitative data. Methods for analyzing talk, text and interaction.

[CIT0008] Tideman M (2008). Resistance, identity of resistance and empowerment among people with intellectual disabilities. Journal of Intellectual Disability Research.

[CIT0009] Turner B. A (1983). The use of grounded theory for the qualitative analysis of organizational behaviour. Journal of Management Studies.

